# The Influence of Economic Factors on the Relationship between Partnership Status and Health: A Gender Approach to the Spanish Case

**DOI:** 10.3390/ijerph19052975

**Published:** 2022-03-03

**Authors:** Jordi Gumà-Lao

**Affiliations:** 1 Department of Political and Social Sciences, Universitat Pompeu Fabra, 08005 Barcelona, Spain; jordi.guma@upf.edu; Tel.: +34-93-542-1969; 2 Sociodemography Research Group (DEMOSOC), Universitat Pompeu Fabra, 08005 Barcelona, Spain

**Keywords:** partnership status, subjective health, economic factors, gender inequalities, Spain

## Abstract

This study explores the relevance of economic factors (e.g., a household’s economic capacity and the prevailing economic context) to understand the relationship between the partnership status and the health of Spanish adult women and men (age 30–59). To do so, it draws on cross-sectional data from the Spanish sample of the European Union Statistics on Income and Living Conditions (EU-SILC) for the years 2005, 2010, and 2015 (i.e., before, during, and after the 2008–2012 economic recession). The results reveal dissimilar patterns of association between partnership status and both the health of, and the economic difficulties faced by Spanish women and men in each of the three years studied. Most notably, the partnership status of Spanish women has a greater impact on their likelihood of experiencing economic difficulties and poor health than does that of their male counterparts. Additionally, women are also more likely to experience economic difficulties during and after the economic recession. The disadvantageous situation of Spanish women in the public sphere is shown to have a negative impact on their ability to cope with the economic difficulties associated with the end of a union and a contextual recession.

## 1. Introduction

The role played by partnership status as a social determinant of health has been extensively examined using both cross-sectional and longitudinal data and by analyzing a variety of health indicators, including mortality, subjective health, and objective health, among others [1,2]. In general, studies find a fairly consistent influence of partnership status on differences in health, with unmarried individuals displaying worse health outcomes [3].

Among the factors that are identified in accounting for the health benefits of living with a partner, this study focuses specifically on those of an economic nature. It appears that a couple benefits from being able to pool their economic resources, with the resulting economies of scale allow for them to optimize their assets [4]. However, the termination of such a union typically results in the disappearance of these material advantages, an outcome that tends to be especially detrimental to the household’s non-breadwinner, more often than not the woman [5]. Therefore, it seems reasonable to hypothesize that the economic context may condition the relationship between partnership status and health, either by increasing the likelihood of those who do not live with a partner of experiencing economic difficulties or by modifying the magnitude of the association between different union situations, and poor health.

The literature indicates that the deterioration in economic conditions in those who have seen their union terminated is considerably more acute in the case of females given the unequal distribution of tasks by gender in the public and private spheres [6]. Unequal gender norms tend to restrict the role of women to the private sphere and create obstacles for their participation in the public sphere (manifesting in lower rates of participation in the labor market, lower wages for the same work, etc.), resulting in their greater economic dependence on their partners [7]. For this reason, a high percentage of women suffer a marked deterioration in their socioeconomic conditions upon ending a union and face considerable difficulties in their efforts to reverse this situation [8]. Therefore, I hypothesized a significant effect of gender inequality in the period analyzed in this paper, which was dependent on the association between partnership status and health.

The literature reports conflicting evidence on the impact of economic recession on overall population health, with findings varying on the specific health outcome under analysis. Some studies have found a negative effect of a period of economic recession on mental health indicators [9,10], while others have reported a neutral or even positive effect on mortality [11]. However, if we consider the economic mechanism underpinning the association between marital status and health, I hypothesized that an adverse economic context increases the likelihood of a person not living with a partner reporting poor health. Moreover, an economic recession is likely to increase the chances of those who previously ended a union of reporting economic hardship, as they must face this change in circumstances from a position of disadvantage.

This paper aimed to shed light on the influence that economic factors exerted on the relationship between partnership status and health. To do so, it adopted a gender perspective in its analysis of the relationship between household economic difficulties, union status and health in different prevailing economic contexts. Two complementary analyses were conducted. First, I examined separately for women and men, the relationship between an individual’s partnership status and self-perceived health in 2005, 2010, and 2015 (that is, before, during, and after the 2008–2012 economic recession) while controlling for a household’s ability to make ends meet. Second, I sook to determine, again separately by sex, whether some partnership statuses were associated with different probabilities of individuals reporting economic hardship. This second analysis complemented the first by providing new insights into the relationship between two factors that are strongly related to health, namely an individual’s partnership status and their economic circumstances [12].

The data analyzed correspond to the Spanish subsample of individuals aged between 30 and 59 included in the European Union Statistics on Income and Living Conditions (EU-SILC) for the years 2005, 2010, and 2015. The analysis was limited to this age range, given the study’s interest in focusing on active individuals at the time of the survey and the fact that this age range is more likely to present one of the partnerships statuses of interest (for details, see below). Self-perceived health was used with the understanding that it captures an integral dimension of health as recommended by the WHO and because of its prevailing validity [13]. This indicator has also been found to be capable of capturing differences in the objective health of a relatively homogenous population, including the adult Spanish population [14].

Three factors account for the particular relevance of the Spanish case: first, the role played by the primary provider of resources and services within Spanish society is more prominent than is typically the case in the rest of Europe [15]; second, the markedly wider gender gap in labor market participation that exists in Spain when compared to its European neighbors, which has consequences on the economic dependence of Spanish women on their partners [16]; and third, the fact that Spain was one of the European countries hit hardest by the 2008–2012 economic recession.

### 1.1. Background

#### 1.1.1. Partnership Status and Health

The effect of registered partnership status (being in a union or not) on health has been clearly demonstrated, with being in a union having a positive impact on health [1,17]. This beneficial effect has been attributed to three basic mechanisms: first, those in a union are subjected to increased control of their risky behaviors and unhealthy habits, both by their partners and society; second, being in a union creates and maintains a social support network beyond that of the immediate household that the couple can fall back on in critical situations; and third, living with a partner can increase material well-being thanks to the combination and optimization of resources through the task specialization of family members.

Logically, the termination of a union implies that the positive influence of these three mechanisms is discontinued in full or in part, resulting in a deterioration of health in the short and medium term. Moreover, the resulting negative impact on health has been shown to be only partially reversed by a new union. That is, the detrimental effects of divorce on social status and well-being seem not to be reversed even with the passage of time and the formation of a new union [18], suggesting that the levels of health reported during the first union are not always recovered [19,20].

Although the termination of a union has been found to reduce the economic resources available to both members of that union, the economic impact is typically reported as being greater for women [21]. The fact that traditional gender norms, to some degree, continue to restrict a woman’s role in the private sphere places women at a disadvantage when terminating a union. Indeed, having to re-enter the labor market has been shown to increase a woman’s likelihood of being paid less than her male counterparts performing the same job [21]. Furthermore, getting divorced presents a greater negative impact on the salary of women who were in work before a breakup, with reductions in income of almost a third, reflecting new constrictions in working hours due to their responsibilities as divorced mothers, regardless of their social stratum. In contrast, this reduction is only observed in men with low salaries [22]. The duration of these negative effects of divorce on the economic status of individuals, while transitory in men, also tends to be chronic in women [23]. This long-term economic impact is evident, for example, in the fact that a higher percentage of divorced women have to extend their working careers, while married women tend to retire earlier [24].

Another registered partnership status that has been shown to have an association with the health of the adult and elderly population is that of being single, although the literature to date presents somewhat conflicting results. On the one hand, health has been reported to be one of the selection mechanisms for individuals in the marriage market, so that poor health or the adoption of unhealthy behaviors (such as alcohol, tobacco or drug use) are associated with a lower probability of initiating and subsequently consolidating a relationship [25,26]. However, it has also been observed that those who have remained single in the long-term present a good capacity to adapt to this situation, unlike those who become single after the breakup of a union. The former, moreover, compensates for the resources they are unable to obtain in the context of a couple and thus improves their mental health [27,28]. However, any greater social integration is limited to a positively selected group of singletons who enjoy such advantages as a high socioeconomic status, which reduces their probabilities of having to face economic hardship [29].

#### 1.1.2. The Effect of Economic Recession on Health

Periods of economic recession have been reported to exert an influence on a population’s health indicators, although results in the literature are somewhat divergent. On the one hand, several studies identified a worsening or, at least, a stagnation in the evolution of such indicators, especially those indicative of mental health (as a consequence of the increase in economic insecurity), the deterioration of working conditions, and an increase in poverty [30,31]. However, other studies reported positive health outcomes including, for example, an improvement in mortality rates as a result of a decrease in occupational accidents and a fall in traffic accidents due to the reduction in commuting [32,33].

#### 1.1.3. Contextual Factors: The Case of Spain

In the case of Western European countries, a number of contextual factors seem likely to exert some influence on the relationship between registered partnership status and health, via elements that impact levels of gender inequality. The specific socioeconomic context and policy interventions (e.g., different levels of state welfare provision) in conjunction with cultural and societal patterns can contribute to either the narrowing or the broadening of the gender gap within the household and, in this way, influence once more the direction and the intensity of the relationship between health and family events. For example, Eikemo et al. [34] stressed the relevance of welfare systems in accounting for some of the differentials in self-perceived health across European countries, with lower levels being found in eastern and southern countries. Despite such reports, Southern Europe has been a subject of little attention in this regard. Yet, the region is of undoubted interest given the specific interplay between family, state and the market in the provision of resources and services related to well-being [35]. As the family and other primary social networks play a more important role in this in Southern Europe, we would also expect these countries to determine health and its associations with partnership status differently than the rest of Europe.

Spain has experienced great, and at the same time rapid, social changes, becoming a more gender egalitarian society; however, it should be borne in mind that the country started from a position of much greater inequality in this respect than most European countries, as a consequence of Franco’s National Catholic dictatorship (1939–1975), which stereotyped the role of women as caregivers [36]. Despite this rapid pace of change towards greater gender equality, the process was impeded by the recent economic recession, in both the public and private spheres, highlighting the importance of the economic context in the evolution of gender inequality in Spain [37,38]. Moreover, the recession hit Spain harder than most other European countries, as evidenced by the fact that the duration of the crisis was longer (from 2008 to 2012), the country’s GDP fell by 7.6%, and its unemployment rate rose alarmingly (from 11% to 26% of the active population) [39].

## 2. Materials and Methods

### 2.1. Samples

The data were drawn from the cross-sectional, Spanish sample of the European Union Statistics on Income and Living Conditions (EU-SILC), administered by Eurostat, for the years 2005, 2010, and 2015 (before, during, and after the 2008–2012 economic crisis). The sample unit in this panel survey was the household and interviews were conducted with individuals aged 16 and over. Each household could be followed up for a maximum period of four years, which means up to 25% of the sample was renewed in each edition of the survey. This accounts for the five-year time lag between each sample in the analysis reported here, aimed at guaranteeing the independence of the three samples. The analysis included only those individuals in the 30–59 age range, providing an original sample of 15,489, 15,958 and 13,961 for the years 2005, 2010, and 2015, respectively. The percentage of individuals that presented complete information for all the variables included in the analysis was 95% for all three years, giving a final working sample of 43,084 (22,403 women and 20,681 men).

In what follows, I provide a more detailed description of the variables analyzed, starting with the dependent variables, followed by the variables of interest, and concluding with the control variables. Given the two complementary analyses described above, the variable “household ability to make ends meet” plays a dual role: as an independent variable in models 1 and 2 and as a dependent variable in models 3 and 4.

#### 2.1.1. Dependent Variables

Self-perceived health: Information obtained from the question “How is your health in general?” (Ranging from “very good”, “good”, “fair”, “poor” to “very poor”). This indicator was chosen on the basis of its proven capacity to inform about a person’s general current health status as well as about any recent changes in that status [40]. It is especially suitable for studying middle-aged populations in which morbidity levels are still low but future health problems are incipient. In line with common practice [41], we grouped the five possible answers into two categories: good health (“good” or “very good” health) and poor health (“fair”, “poor” or “very poor” health).

Household ability to make ends meet: To account for the economic capacity of the entire household a subjective assessment was taken of a household’s economic situation. The original answers were operationalized as follows: “with great difficulty”, “with difficulty”, and “with some difficulty” were operationalized as “with difficulties” (1), and “fairly easily”, “easily”, and “very easily” were operationalized as “easily” (0).

#### 2.1.2. Variables of Interest

Partnership status: Information obtained from combining two questions: one, concerning the current union status of the interviewee and a second, as to whether he or she is in a consensual union. The variable comprises the following three categories: single (neither married nor in a consensual union); in a union (married or in a consensual union); and separated, widowed, or divorced.

Period: a variable identifying the year in which the survey was conducted (2005—before the economic recession, 2010—during the recession, and 2015—after the recession).

#### 2.1.3. Control Variables

Education: the highest educational level attained by the interviewees grouped into three categories: low (ISCED levels 0 to 2); medium (ISCED levels 3 and 4); and high (ISCED levels 5 and 6).

Children: Living with a child or children aged 16 or younger. This final variable was dichotomous (either living with or without children aged 16 or younger).

Age: the age of the interviewees was included in its continuous form.

Economic status: Information recorded in accordance with the interviewees’ self-defined economic status. Original responses were operationalized as a final variable with four categories: working (full- or part-time), unemployed, homemaker, or other (i.e., student, in further training, unpaid work experience, retired/taking an early retirement/left work, doing compulsory military or community service, and other inactive persons). Those who declared themselves as being permanently disabled and/or unfit to work were excluded from the analysis due to an obviously strong relationship with poor health status.

### 2.2. Statistical Analysis

A logistic regression was used to model the probabilities of individuals declaring both poor health status (models 1 and 2 for women and men, respectively) and difficulties making ends meet (models 3 and 4 for women and men, respectively). In models 1 and 2, the probabilities of reporting poor self-perceived health were calculated according to the interaction between partnership status and the year in which the survey was conducted and in models 3 and 4, the probabilities of reporting problems to make ends meet were calculated according to the interaction between partnership status and the year in which the survey was conducted. Thus, the first of these calculations assessed whether the degree of probability of being in poor health for the three partnership statuses reported is dependent on the prevailing economic situation, while the second of these calculations determined whether those not living with a partner present a higher probability of reporting experiencing economic difficulties and whether this probability is dependent on the prevailing economic situation (i.e., pre, during, and post economic crisis).

Separate models were calculated for women and men to capture a possible gendered effect in the multivariate analysis. To facilitate interpretation of the regressions I report, separately by gender, the probabilities of a household facing difficulties to make ends meet and of being in poor health for the specific combination of variables of interest outlined above. In both cases, the regressions including all the control variables were calculated for 95% confidence intervals (complete estimates for all models are reported in Supplementary Tables S1–S4).

## 3. Results

### Descriptive

Table 1 shows the characteristics of the Spanish sample obtained from the EU-SILC for individuals aged between 30 and 59 for the years 2005, 2010, and 2015. The table, which reports the variables included in the analysis for women and men separately, shows marked differences between the profiles of both sexes in two of the three main variables: self-perceived health and partnership status. In the case of health, results confirm the worse health profile of Spanish women, as described previously in practically all Western European countries. In the case of partnership status, while similar percentages of women and men are in a union, a higher percentage of women are separated, divorced, or widowed and a higher percentage of men are single. However, in the case of the third variable, the subjective perception of the ability to make ends meet for the household as a whole, women and men present very similar values; however, a notable increase is recorded in individuals of both sexes reporting difficulties in 2010 and 2015.

Results show an increase in the share of the population reporting a high level of education over the period studied, confirming the expansion of education in Spain, especially in regard to women. Yet, results in relation to employment status indicate the persistence of traditional gender norms, with higher percentages of employment in men and residual values of exclusive dedication to household tasks being higher in women. However, there has been a progressive decline in the percentage of Spanish women aged between 30 and 59 who report an exclusive dedication to domestic tasks in the period studied. Finally, the mean age of the two sexes is similar in the three years of the survey reported here.

**Figure 1:**
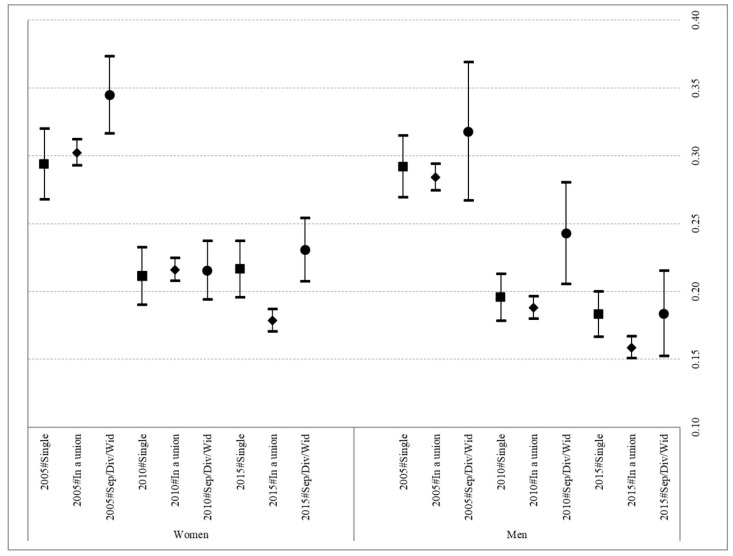
Predicted probabilities of poor health according to partnership status and year in Spanish women and men aged 30–59. 2005, 2010 and 2015. Source: Spanish sample EU-SILC 2005, 2010 and 2015. Note: Both models control for education, employment status, capacity to make ends meet, living with children < 16 and age.

Figure 1 shows the probabilities of respondents declaring themselves to be in poor health (models 1 and 2). In general, the results confirmed a greater probability of poor health in women. Moreover, in the first period (2005–2010), a downward trend was detected in these probabilities; however, this trend was interrupted for both sexes in the period from 2010 to 2015. In the case of women, a fairly clear pattern emerged, with significant differences observed in 2005 and 2015 between those in some type of union at the time of the interview and those who had experienced the end of a union, with the latter presenting a higher probability of poor health. However, this difference was not evident in 2010. In the case of men, significant differences were only observed between those in a union and those having suffered the end of a union in 2010, in the middle of the economic crisis.

Finally, the ability of the households to make ends meet also showed a significant association with health, with those facing economic difficulties presenting greater probabilities of poor health than those not reporting any economic difficulties, regardless of sex (see Table S1 for the coefficients of these two models).

**Figure 2:**
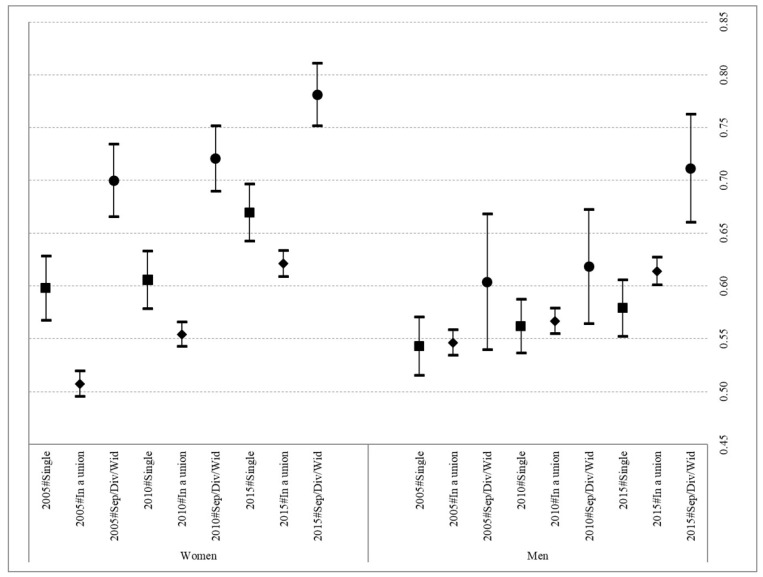
Predicted probabilities of having difficulties to make ends meet according to partnership status and year in Spanish women and men aged 30–59. 2005, 2010 and 2015. Source: Spanish sample EU-SILC 2005, 2010 and 2015. Note: Both models control for education, employment status, age, and living with children under the age of 16.

Figure 2 shows the probabilities of respondents declaring themselves as having difficulties to make ends meet by sex (models 3 and 4, also see Table S2 for the coefficients of these two models). The results highlight clear gendered differences in the likelihood of reporting such difficulties, with the highest values being reported by women who do not live with a partner. In fact, within women, there is a growing tendency over time to report facing financial difficulties of this kind, regardless of their partnership status, while in men the same is only true in those that have ended a union and those who were interviewed in 2015.

Clear gendered differences are again apparent in relation to the partnership status of the respondents. In women, the gradient is clear: those who are in some type of union are in the strongest position, followed by singletons, and then those having experienced the end of a union in last. However, in men, significant differences are only observed in the case of those interviewed in 2015 who had experienced the end of a union, as noted above.

## 4. Discussion

This study explores the relevance of economic factors (both a household’s economic capacity and the prevailing economic context of the country) in understanding the relationship between partnership status and health in Spanish adult women and men (aged 30 to 59) using cross-sectional data from the EU-SILC survey for the years 2005, 2010, and 2015 (that is, before, during, and after the 2008–2012 economic recession). The results obtained contribute to furthering our understanding of the importance of these economic factors in accounting for the association between partnership status and health from a gender perspective.

First and foremost, the results confirm the relevance of gender in the relationship between partnership status and health. In the case of women, this relationship was found to be quite clearly significant (with the sole exception of the year 2010) in the context of a severe international economic recession. This outcome would seem to indicate that, at its peak, the effect of the crisis impacted all partnership statuses, thus reducing the importance of certain social determinants of health for understanding the overall health of the population. In contrast, significant differences were only observed in men in the last year of the study (2015), when men who had experienced the end of a union presented a higher probability of poor health than those in some kind of union. As such, the results confirm the findings of previous studies that report the greater importance to of marital status as a social determinant of health to Spanish adult women than to their male counterparts [42].

Finally, the study reports a general stagnation in the previous trend towards better health at the population level before the 2008–2012 economic recession in Spain, regardless of partnership status. As discussed, it seems that the negative effect of the economic crisis was not immediate; rather, it has become more evident in the post-crisis period, as a result of the increase in the share of the Spanish population presenting a more vulnerable socioeconomic profile which, in turn, has impacted its health profile [43]. This finding is in line with the reported increase in the probability of Spanish adults declaring themselves as facing economic difficulties both during and after the economic recession. Moreover, in the case of women, this increase in the probability of having difficulties making ends meet each month presents a clear gradient with their partnership status. Women who experienced the breakup of their previous union and were not living with another partner at the time of being interviewed presented a higher probability of suffering with economic difficulties at all three observation dates. However, this gradient with partnership status was not evident in men, as no differences in the probability of facing economic difficulties were detected. This can be interpreted as the outcome of the continuing importance of traditional gender norms and their consequent role as breadwinners, so that when men leave a union they tend to maintain or only slightly reduce their economic capacity. In contrast, their former female partners, who tended to be economically dependent on them in the past, experienced a dramatic deterioration in their economic circumstances. Moreover, as the results in Tables S1 and S2 show, having to face these financial difficulties increases the probability of reporting poor health in all cases.

In the light of these results, it is clear that women are much more exposed to the negative effects derived from situations that might impact their financial circumstances, such as the end of a union or an economic recession. Although decreasing progressively, the economic dependence of women on their partners as a result of their lower participation in the labor market leaves them vulnerable to higher levels of precariousness, especially in the case of those terminating a union. Traditional gender norms that endow men with greater prominence in the public sphere, while restricting women to the private sphere, have been shown to increase female vulnerability in the event of divorce or the termination of a union. Having to abandon their professional careers, or to put them on hold, in order to devote their time to domestic duties places women in a position of economic subordination with respect to their partners. Additionally, in the case of those women who remain in the labor market, they often find themselves forced by social norms to place their professional careers in subordinate to family obligations, thereby limiting their opportunities for professional consolidation and promotion, therefore reducing their earning power [44]. In short, traditional gender norms place women at a disadvantage as they seek to recover from the negative consequences of an economic recession or the termination of a union. Moreover, this increases the probability of women reporting poor self-perceived health after the end of a union and suffering the effects of a general reduction in health improvements associated with an economic recession. These outcomes are in line with previous research that stresses the disadvantageous social position of women as the main driver of their greater propensity to face higher probabilities of poor health [45] and, as such, contributes to explaining the survival-health paradox [46].

This study has a number of limitations. Having access to information on the exact timing of the termination of a union would have facilitated a better assessment of the resilience of both women and men, as it would have been possible to assess whether the negative effects of such an event disappear or remain unchanged over time. A further limitation is related to the cross-sectional nature of the data analyzed, as it does not indicate whether the economic hardships reported started before or after the end of a union. This limits any interpretation of our results to associations and prevents the application of other types of analyses that could disentangle the direct and indirect effects of facing economic difficulties on health. Related to this, having information on the time elapsed since the end of the couple’s relationship would improve the understanding of the mechanisms underlying the results obtained. Finally, the results presented are only valid for the specific case of the Spanish population, and any future research should seek to incorporate other European countries so as to assess whether the outcomes reported here are generalizable or whether the different levels of gender inequality experienced in each country affect the pattern of results.

## 5. Conclusions

Clearly, the promotion of gender-equal norms and attitudes in both the public and private sphere, by means of the implementation of equality policies, would contribute to increasing the level of resilience of women in situations that might lead to economic hardship. This greater resilience would translate into a reduction in the levels of economic inequality in the population as a whole and would also contribute to improving the health profile of the female population. Moreover, it is not only female health that would benefit, since it has been shown that men who live with women with a high socioeconomic level present better health indicators than their similar counterparts with partners that have a less advantageous socioeconomic profile [47].

## Figures and Tables

**Table 1:** Characteristics of the working sample, ages 30–59 (%). Spain 2005, 2010 and 2015.

		Women			Men		
		2005	2010	2015	2005	2010	2015
Household capacity to make ends meet	Easily	43.7	43.06	37.97	45.03	43.18	39.28
	Difficulty	56.3	56.94	62.03	54.97	56.82	60.72
	Total	100	100	100	100	100	100
Self-perceived health	Good	72.96	79.44	80.9	76.39	83.28	84.22
	Poor	27.04	20.56	19.1	23.61	16.72	15.78
	Total	100	100	100	100	100	100
Union status	Single	11.59	13.84	14.44	16.51	19.42	20.1
	In a union	79.79	76.71	75.73	80.62	76.61	75.15
	Sep/Div/Wid	8.62	9.45	9.83	2.87	3.97	4.75
	Total	100	100	100	100	100	100
Education	Low	52.26	43.06	37.88	50.3	45.46	42.37
	Medium	21.29	22.48	22.94	22.99	23.22	24.33
	High	26.44	34.47	39.18	26.71	31.32	33.3
	Total	100	100	100	100	100	100
Employment status	Empoyed	55.18	62.15	62.01	88.98	81.19	78.55
	Unemployed	9.32	15.7	21.5	6.86	15.83	19.62
	Fulfilling domestic tasks	32.61	20.07	15.02	0.13	0.07	0.08
	Other situation	2.88	2.07	1.47	4.03	2.9	1.75
	Total	100	100	100	100	100	100
Average age		43.8	44.3	45.5	43.8	44.1	45.3
*N*		7669	7776	6958	7068	7231	6382

Source: Spanish sample EU-SILC 2005, 2010 and 2015.

## Data Availability

The data used in this research come from anonymized datasets of population surveys run by European Statistical Institutes and delivered to researchers upon request by EUROSTAT.

## Supplementary Materials

The following supporting information can be downloaded at: https://www.mdpi.com/article/10.3390/ijerph19052975/s1, Table S1. Coefficients of logistic regression models about poor health according to partnership status and year among Spanish women aged 30–59. 2005, 2010 and 2015. Table S2. Coefficients of logistic regression models about poor health according to partnership status and year among Spanish men aged 30–59. 2005, 2010 and 2015. Table S3. Coefficients of logistic regression models about having difficulties to make ends meet according to partnership status and year among Spanish women aged 30–59. 2005, 2010 and 2015. Table S4. Coefficients of logistic regression models about having difficulties to make ends meet according to partnership status and year among Spanish men aged 30–59. 2005, 2010 and 2015.
