# Supplementary material for: The Influence of Economic Factors on the Relationship between Partnership Status and Health: A Gender Approach to the Spanish Case
Source: Int J Environ Res Public Health. 2022 Mar 3;19(5):2975. doi: 10.3390/ijerph19052975 (PMC8910377; doi:10.3390/ijerph19052975)
Supplement: Supplementary file 1 [file ijerph-19-02975-s001.zip › ijerph-1539131-supplementary.pdf]

**Table S1. Coefficients of logistic regression models about poor health according to partnership status and year among Spanish women aged 30-59. 2005, 2010 and 2015.**

| | | Coef. | Std. Err. | Sign. |
|---|---|---|---|---|
| **Year of the survey#partnership status (Ref: 2005#In a union)** | 2005#Single | 0.05 | 0.09 | |
| | 2005#Sep/Div/Wid | 0.28 | 0.09 | ** |
| | 2010#Single | -0.32 | 0.09 | ** |
| | 2010#In a union | -0.35 | 0.05 | *** |
| | 2010#Sep/Div/Wid | -0.15 | 0.09 | |
| | 2015#Single | -0.19 | 0.09 | * |
| | 2015#In a union | -0.60 | 0.05 | *** |
| | 2015#Sep/Div/Wid | -0.16 | 0.09 | ** |
| **Economic difficulties (Ref: Not having)** | Having | 0.61 | 0.04 | *** |
| **Education (Ref: Low)** | Medium | -0.34 | 0.04 | *** |
| | High | -0.66 | 0.05 | *** |
| **Employment status (Ref: Employed)** | Unemployed | 0.30 | 0.05 | *** |
| | Fulfilling domestic task | 0.20 | 0.04 | *** |
| | Other situations | 0.85 | 0.10 | *** |
| **Living with children (Ref: Not living)** | Living with children | 0.07 | 0.13 | |
| **Age** | | 0.06 | 0.00 | *** |
| **Constant** | | -3.97 | 0.12 | *** |
| **Pseudo R-Square** | | 0.10 | | |

Source: Spanish sample EU-SILC 2005, 2010 and 2015

*** <0,001; **<0,01; *<0,05

**Table S2. Coefficients of logistic regression models about poor health according to partnership status and year among Spanish men aged 30-59. 2005, 2010 and 2015.**

| | | Coef. | Std. Err. | Sign. |
|---|---|---|---|---|
| **Year of the survey#partnership status (Ref: 2005#In a union)** | 2005#Single | 0.18 | 0.08 | * |
| | 2005#Sep/Div/Wid | 0.29 | 0.16 | |
| | 2010#Single | -0.39 | 0.09 | *** |
| | 2010#In a union | -0.50 | 0.05 | *** |
| | 2010#Sep/Div/Wid | -0.13 | 0.14 | |
| | 2015#Single | -0.44 | 0.09 | *** |
| | 2015#In a union | -0.68 | 0.05 | *** |
| | 2015#Sep/Div/Wid | -0.49 | 0.15 | ** |
| **Economic difficulties (Ref: Not having)** | Having | 0.47 | 0.04 | *** |
| **Education (Ref: Low)** | Medium | -0.25 | 0.05 | *** |
| | High | -0.53 | 0.05 | *** |
| **Employment status (Ref: Employed)** | Unemployed | 0.47 | 0.05 | *** |
| | Fulfilling domestic task | 0.81 | 0.51 | |
| | Other situations | 1.15 | 0.09 | *** |
| **Living with children (Ref: Not living)** | Living with children | 0.04 | 0.15 | |
| **Age** | | 0.06 | 0.00 | *** |
| **Constant** | | -4.04 | 0.13 | *** |
| **Pseudo R-Square** | | 0.08 | | |

Source: Spanish sample EU-SILC 2005, 2010 and 2015

*** <0,001; **<0,01; *<0,05

**Table S3. Coefficients of logistic regression models about having difficulties to make ends meet according to partnership status and year among Spanish women aged 30-59. 2005, 2010 and 2015.**

| | | Coef. | Std. Err. | Sign. |
|---|---|---|---|---|
| **Year of the survey#partnership status (Ref: 2005#In a union)** | 2005#Single | 0.42 | 0.08 | *** |
| | 2005#Sep/Div/Wid | 0.93 | 0.10 | *** |
| | 2010#Single | 0.46 | 0.07 | *** |
| | 2010#In a union | 0.22 | 0.04 | *** |
| | 2010#Sep/Div/Wid | 1.05 | 0.09 | *** |
| | 2015#Single | 0.77 | 0.08 | *** |
| | 2015#In a union | 0.53 | 0.04 | *** |
| | 2015#Sep/Div/Wid | 1.41 | 0.10 | *** |
| **Education (Ref: Low)** | Medium | -0.61 | 0.04 | *** |
| | High | -1.34 | 0.04 | *** |
| **Employment status (Ref: Employed)** | Unemployed | 0.96 | 0.05 | *** |
| | Fulfilling domestic task | 0.29 | 0.04 | *** |
| | Other situations | 0.15 | 0.10 | |
| **Living with children (Ref: Not living)** | Living with children | 0.21 | 0.11 | * |
| **Age** | | -0.03 | 0.00 | *** |
| **Constant** | | 1.69 | 0.10 | *** |
| **Pseudo R-Square** | | 0.11 | | |

Source: Spanish sample EU-SILC 2005, 2010 and 2015

*** <0,001; **<0,01; *<0,05

**Table S4. Coefficients of logistic regression models about having difficulties to make ends meet according to partnership status and year among Spanish men aged 30-59. 2005, 2010 and 2015.**

| | | Coef. | Std. Err. | Sign. |
|---|---|---|---|---|
| **Year of the survey#partnership status (Ref: 2005#In a union)** | 2005#Single | -0.02 | 0.07 | |
| | 2005#Sep/Div/Wid | 0.27 | 0.16 | |
| | 2010#Single | 0.07 | 0.07 | |
| | 2010#In a union | 0.10 | 0.04 | * |
| | 2010#Sep/Div/Wid | 0.34 | 0.14 | * |
| | 2015#Single | 0.15 | 0.07 | * |
| | 2015#In a union | 0.32 | 0.04 | *** |
| | 2015#Sep/Div/Wid | 0.81 | 0.14 | *** |
| **Education (Ref: Low)** | Medium | -0.57 | 0.04 | *** |
| | High | -1.26 | 0.04 | *** |
| **Employment status (Ref: Employed)** | Unemployed | 1.31 | 0.06 | *** |
| | Fulfilling domestic task | -0.64 | 0.49 | |
| | Other situations | 0.02 | 0.09 | |
| **Living with children (Ref: Not living)** | Living with children | 0.09 | 0.15 | |
| **Age** | | -0.02 | 0.00 | *** |
| **Constant** | | 1.39 | 0.10 | *** |
| **Pseudo R-Square** | | 0.10 | | |

Source: Spanish sample EU-SILC 2005, 2010 and 2015

*** <0,001; **<0,01; *<0,05
